# Development and evaluation of an intelligent centralized transportation dispatching information system in a large tertiary hospital: a single-center retrospective observational study

**DOI:** 10.3389/fpubh.2026.1949019

**Published:** 2026-10-09

**Authors:** Mengjiao Zhang, Jin Xue, Rui Shi

**Affiliations:** Department of Information and Data Management, West China Hospital, Sichuan University, Chengdu, Sichuan, China

**Keywords:** central transportation, healthcare quality management, intelligent dispatching system, internet of things, radio-frequency identification, retrospective study, smart hospital

## Abstract

**Background:**

Traditional hospital central transportation adopts manual telephone dispatch, bringing high communication costs, poor traceability, mismatched resources and weak quality control, which impede clinical efficiency and patient safety. This study designs an intelligent dispatching system integrating RFID, IoT and mobile terminals, and assesses its performance in a large tertiary hospital.

**Methods:**

A single-center retrospective observational study was carried out at West China Hospital. The system combines RFID-IoT real-time positioning and multi-objective scheduling algorithms, with two-way connections to HIS, examination platforms and PDAs. We analyzed system data of transport tasks, time metrics, tool utilization and patient satisfaction from Jan 1 to Jun 30, 2026 (post-implementation). Pre-2025 non-digital records were only used for qualitative reference; incomplete raw data excluded formal statistical comparison and inter-period hypothesis testing. Continuous variables were median (interquartile range); categorical variables *n* (%).

**Results:**

Over 6 months, the system handled four core transport scenarios. Post-implementation median single-task dispatch time reached 0.57 (0.31–0.82) min, and emergency examination transport time 24.3 (13.41–32.87) min. Among 24,545 tool records, independent ambulation (54.5%) and wheelchairs (32.0%) made up 86.5% of transports, stretchers (6.5%) and hospital beds (7.0%) for critical and postoperative patients accounted for the rest 13.5%. In 5,508 surveyed inpatients, overall transport satisfaction hit 97.78%, with item-level satisfaction rates ranging from 95.51% (staff attentiveness) to 98.14% (staff appearance/professional grooming).

**Conclusion:**

This single-center retrospective evaluation shows the RFID/IoT intelligent dispatching system achieves brief dispatch & transport durations and high patient satisfaction. Without matched complete pre-implementation baseline data, results only describe post-launch performance instead of causal improvement evidence. Prospective multi-center or interrupted-time-series studies with standardized baselines are required to verify its incremental value.

## 1 Introduction

The hospital central transportation system is a critical logistical hub connecting clinical care, diagnostic and therapeutic services, and logistical support, and is responsible for patient escort, specimen transport, medication delivery, and material dispatch. As hospitals expand in scale and service volume, the complexity of transportation tasks increases accordingly. Traditional dispatch models based on telephone calls and two-way radios have well-recognized limitations: task assignment is subjective, information transfer is prone to distortion, end-to-end tracking is largely absent, and performance evaluation lacks objective data support. These limitations can lead to misallocation of transport resources and inefficiency, and—more importantly—may contribute to safety events such as specimen mis-delivery, transport delays, or patient falls (1–6). Given that RFID and IoT technologies are increasingly used to improve efficiency and safety in medical specimen management and logistics (7–9).

In recent years, 5G, IoT, and RFID technologies have provided technical support for the digital transformation of hospital logistics. Intelligent logistics systems that combine sensors, RFID tags, positioning systems, and computing infrastructure have been proposed as a way to enhance system-level decision-making and execution, and empirical evaluations in real hospital settings have begun to demonstrate operational benefits. For example, a real-time IoT-based positioning and data-driven dispatch system implemented at a Taiwanese hospital (10) reduced overtime delays in patient transport from 41.0% under traditional dispatch to 26.5% under the new system. Similarly, autonomous mobile robots and RFID-based tracking systems have been piloted for material, medication, and patient transport to reduce manual material handling burden and increase time available for direct patient care (7, 8, 11, 12). However, existing literature predominantly focuses on a single transport scenario (e.g., patient transport alone or specimen transport alone), and systematic, large-scale empirical evidence on multi-scenario, integrated intelligent dispatching platforms deployed across an entire large tertiary hospital remains limited.

This study reports the design and functional architecture of an intelligent centralized transportation dispatching information system deployed at West China Hospital, Sichuan University, integrating RFID, real-time location tracking, and an intelligent scheduling algorithm. Using a retrospective, single-arm, descriptive analytic approach, we characterize the system’s operational performance across four dimensions—transport efficiency, task-scenario workflow, resource/tool utilization, and patient satisfaction—during a defined six-month post-implementation period, with the aim of providing a practical reference for the digital transformation of logistics services in comparable tertiary hospitals (13–15).

## 2 Materials and methods

### 2.1 Study design and setting

This was a single-center, retrospective, observational study conducted at West China Hospital, Sichuan University, a large tertiary academic medical center with 4,900 open beds, more than 200,000 annual inpatient admissions, and a dedicated central transportation workforce of 334 full-time staff processing more than 20,000 transportation tasks per day.

The intelligent centralized transportation dispatching information system was progressively deployed at the hospital beginning in 2020. The analytic dataset for this report comprises all transportation tasks, adverse-event records, complaint records, and satisfaction survey data captured by the system between January 1, 2026 and June 30, 2026 (“post-implementation period”). Records generated during periods of system malfunction were excluded as invalid.

A note on the pre-implementation comparison. Because the central transportation department did not maintain a standardized digital record prior to system deployment, no formally matched pre-implementation control dataset with a clearly defined observation window was available. Historical operating conditions before 2020 are described in the Discussion in qualitative and, where available, approximate quantitative terms drawn from non-systematic paper logs and staff recollection; these figures are presented for descriptive/contextual purposes only, are not subjected to inferential statistical testing against the post-implementation dataset, and should not be interpreted as a rigorously sampled control arm. We consider this absence of a well-defined baseline dataset to be an important limitation of the present study (see Section 4.6), and we recommend that future work adopt a pre-specified interrupted-time-series or prospective concurrent-control design.

### 2.2 System architecture and functional design

The intelligent centralized transportation dispatching system operates within the hospital’s internal network and integrates four core technical components (Figure 1):

1. Multi-platform interoperability. Bidirectional data interfaces with the Hospital Information System (HIS), the examination/appointment platform, the Laboratory Information System (LIS), and handheld PDA terminals automatically synchronize physician orders, appointments, specimen information, and staff location data, eliminating manual data-entry error.
2. RFID–IoT closed-loop tracking. The system combines RFID identification with IoT-based sensing to construct a closed-loop, end-to-end traceable management framework. PDA terminals and IoT positioning modules enable real-time localization and trajectory tracking of transport staff. RFID tagging supports real-time tracking of transport personnel and material location, with automatic alerts triggered for overdue tasks, ensuring consistency among “person, item, and order” and producing a fully electronic, auditable task record.
  - Hardware components, Commercially available off-the-shelf hardware is adopted, including handheld PDA terminals integrated with 1D/2D barcode readers and UHF RFID readers supporting the 920 MHz–925 MHz ultra-high frequency band; passive UHF RFID tags mounted on transport equipment and specimen carriers; and fixed RFID readers deployed at key handover nodes. When personnel pass through the coverage area of a node, the reader identifies the tag on the transport equipment and uploads the tag ID, reading timestamp and device location information to the platform. The system determines the area where the personnel are located based on the predefined geographic coordinates of the reader. The deployed hardware comprises Maifurui (麦孚瑞) M61 handheld PDA terminals and Gomrit G6002 fixed RFID readers.
3. Intelligent scheduling algorithm. A dedicated scheduling engine integrates historical transport-task data, departmental spatial distribution, real-time staff location, current workload, and task urgency to generate dispatch decisions dynamically. Three complementary algorithmic components are used:

- Urgency-weighted priority assignment, which assigns tasks a dispatch-priority score as a function of clinical urgency, order type, and waiting time, so that time-critical tasks (e.g., emergency examinations) are automatically escalated to the highest priority tier;
- Route-based task clustering/matching, which identifies spatially and temporally compatible tasks (e.g., multiple examinations for the same patient, or tasks along a common route) and merges them into a single dispatch to reduce redundant trips; and
- Fallback re-assignment for unclaimed tasks, in which an initial task is first opened to all available staff for voluntary claiming; if unclaimed after a defined time window, the system automatically re-assigns the task to an available on-duty staff member to prevent task stagnation.

**Figure 1:**
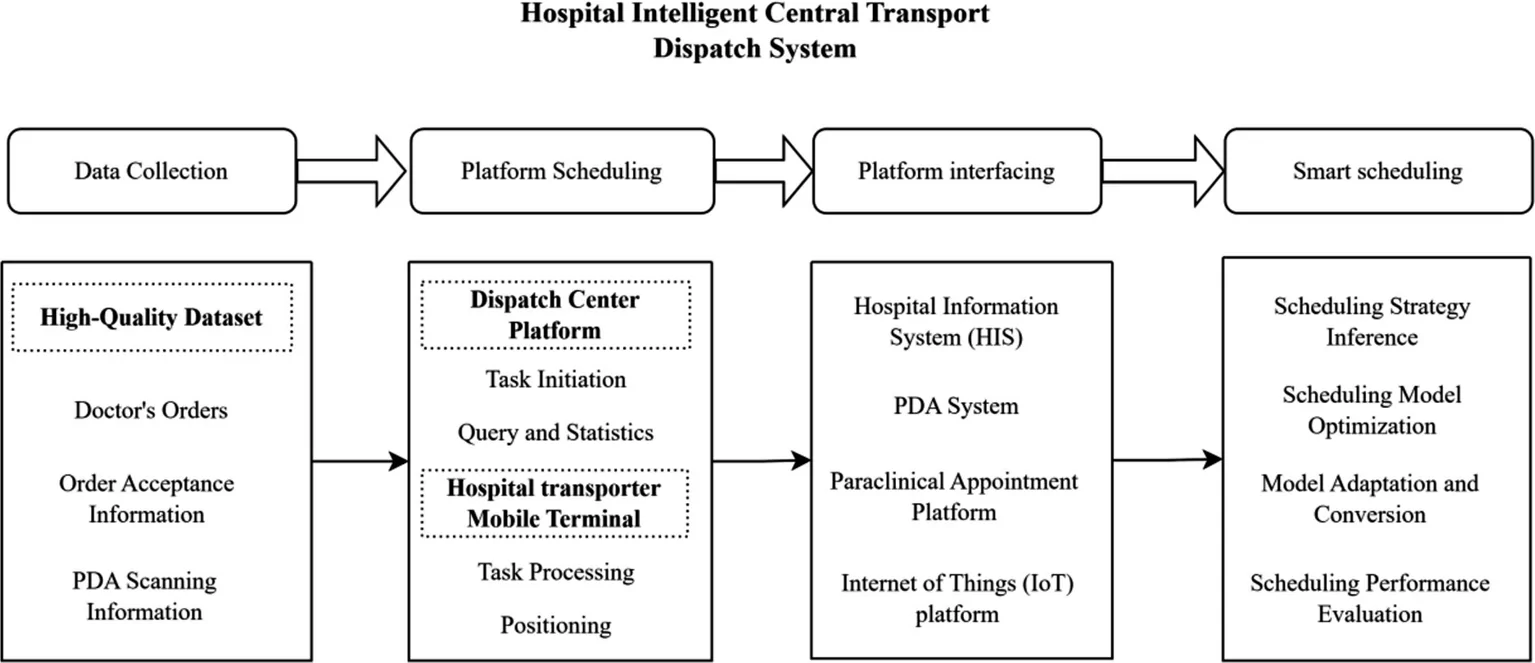
Schematic architecture of the hospital intelligent central transportation dispatching system, illustrating the full workflow covering data acquisition, centralized scheduling, system interconnection, and intelligent optimization.

A closed-loop operational-feedback mechanism continuously collects response time, transport duration, staff workload, and task-completion quality for each dispatch, and uses these data to iteratively refine scheduling-rule parameters, with the goal of improving dispatch accuracy, route efficiency, and overall transport throughput over time.

Algorithm 1 Core dispatch decision logic

The deployed dispatch engine uses a rule-based weighted scoring framework (Schemes A and B) with a group-dispatch fallback (Scheme C). The following description reflects the technical algorithm specification supplied for this study; configurable values are reported as deployed defaults rather than illustrative examples.

Input: task t; on-duty candidate staff; staff online/idle status; current location; current workload; idle duration; staff level; order urgency; and route/point-location information.

1. Pre-filtering: exclude staff who are off duty/offline or who meet the configured urgent-task load limit. The remaining candidates are ranked using the active scoring scheme.
2. Scheme A (baseline weighted scoring): ONLINE 0.30; IDLE 0.20; NEAR_START 0.20; LOAD 0.15; IDLE_DURATION 0.10; LEVEL 0.05. The composite score is the weighted sum of the enabled dimensions.
3. Scheme B (route/load-enhanced scoring): ONLINE 0.25; IDLE 0.15; NEAR_START 0.10; ROUTE 0.25; LOAD 0.20; IDLE_DURATION 0.05; LEVEL 0.05. DIRECTION is an optional additional dimension (0.05) when enabled; weights are normalized to sum to 1.0.
4. Assignment rule: if only one eligible candidate remains, assign directly. If ≥2 candidates remain, assign the highest-scoring candidate when the top score is ≥60 and the top-to-second score difference is ≥5; otherwise enter Scheme C group dispatch.
5. Scheme C fallback: open the task to eligible staff for voluntary claiming. The system scans every 1 min; after 3 min without a claim, it refreshes candidates, re-runs Scheme A/B with fallback thresholds (minimum score 50; minimum lead 3), and forcibly assigns the best candidate. A task can undergo at most one fallback force-assignment; if selection still fails, the task is escalated to manual follow-up.
6. Urgent-order adjustment: for emergency/urgent tasks, the relevant dimension weights are multiplied by predefined correction factors and renormalized; the normal passing threshold is reduced from 60 to 55. The technical specification should be cited as supplementary material if the journal permits.

Output: task-to-staff assignment and, when applicable, group-dispatch/fallback status.

### 2.3 Workflow across four Core transportation scenarios

The system supports four core scenarios (Figure 2): patient examination transport, clinical specimen transport, medication delivery, and material/personnel dispatch.

1. Patient examination transport. Standardized interfaces integrate HIS physician orders with the examination-appointment platform for real-time synchronization and automated classification. Emergency orders are automatically assigned the highest dispatch weight via the urgency-weighted algorithm, enabling near-immediate (“second-level”) priority dispatch. For routine (non-emergency) orders, the system integrates appointment timing, departmental spatial distribution, and patient characteristics to identify optimal transport windows, and uses route-based clustering to merge multiple examinations for the same patient into a single transport trip, reducing redundant movement.
2. Clinical specimen transport. A barcode-based digital tracking system governs specimen handling. For emergency specimens, on-site barcode scanning immediately triggers extraction of specimen type, destination department, urgency level, and delivery deadline, generating a dedicated, time-limited work order with priority access to transport capacity. Routine specimens are batch-dispatched according to departmental geographic distribution and collection time windows using the route-matching algorithm, which plans an optimized multi-stop circuit. A bidirectional “dispatch–transport–receipt” barcode verification protocol is applied throughout, with all operational data uploaded in real time to support a traceable digital audit trail intended to reduce the risk of specimen mismatch, loss, or delay.
3. Medication transport. Integration with the pharmacy dispensing system enables a barcode-triggered, fully logged delivery workflow. After pharmacy verification and dispensing scan, the system automatically extracts medication category, destination ward, patient identifier, and dispensing time to generate a transport order without manual entry. Time-stamped electronic records are maintained at pickup, transport, and delivery/receipt, with differentiated scheduling logic for routine versus emergency medications.
4. Material and personnel dispatch. An integrated “self-service request + cloud-based intelligent scheduling” model allows clinical nursing staff to submit material-delivery or patient-escort requests via mobile terminal, replacing offline reporting and telephone dispatch. The system combines real-time staff location, duty status, and workload with the route-matching algorithm to identify and assign the nearest available staff member. If an initial assignment fails or receives no response, the fallback re-assignment algorithm is triggered as described in Section 2.2. Identity verification—via wristband barcode scanning for patients and staff—is integrated into the handoff process.

**Figure 2:**
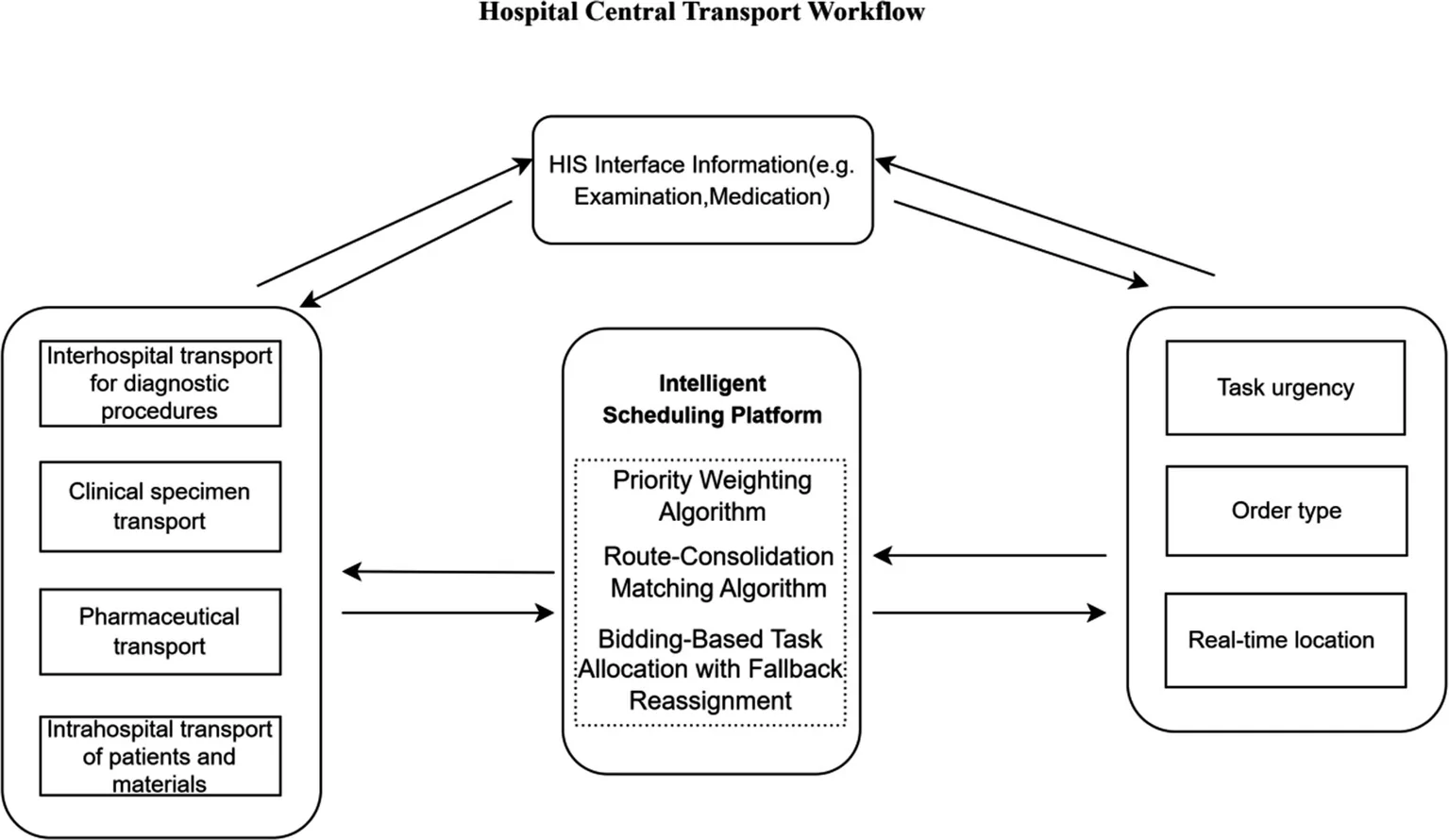
Schematic workflow of the centralized hospital transport management platform, illustrating task generation from the HIS via surgical, medication, and examination interfaces, and coordination via the intelligent scheduling platform.

### 2.4 Outcome measures

1. Efficiency indicators: median single-task dispatch time (minutes); median emergency-examination transport time (minutes); time-of-day task-volume distribution; daily volume of telephone-based (manual) dispatch requests.
2. Operational indicators: distribution of tasks by time-of-day category; distribution of tasks by transport-tool type (independent ambulation, wheelchair, stretcher, bed transfer).
3. Satisfaction indicators: a standardized inpatient satisfaction questionnaire (0–100-point scale per item), covering staff appearance (professional grooming and uniform/identification-badge compliance), staff attitude, identity-verification practice, staff attentiveness, responsiveness to questions, and assistance provided; satisfaction was additionally summarized as the proportion of respondents scoring ≥ 80 points (“satisfied”) on the overall item.

### 2.5 Statistical analysis

Data were analyzed using SPSS version 26.0. Continuous variables are reported as median (interquartile range, IQR), and categorical variables as *n* (%). Because no standardized, matched pre-implementation dataset was available, no inferential before–after hypothesis testing was performed. Any within-post-implementation comparisons, if reported elsewhere, should be explicitly identified and justified.

### 2.6 Ethics statement

This study analyzed retrospective, de-identified, aggregate operational data generated during routine hospital logistics management (transport-task logs, timestamps, and anonymized satisfaction-survey summaries); it did not involve identifiable patient health information, individual-level clinical outcomes, or any change to clinical care, and no individual patient consent process was applicable to the analysis performed. Consistent with widely used frameworks for distinguishing quality-improvement activity from human-subjects research—under which quality assurance and quality-improvement studies, program evaluation, and performance reviews used exclusively for assessment, management, or improvement purposes are not considered research requiring ethics-board review.

## 3 Results

### 3.1 Transportation efficiency

During April 2026, the system processed approximately 46,000 emergency examinations and 69,000 routine examinations. Over the full post-implementation observation window, the median single-task dispatch time was 0.57 (0.31–0.82) minutes, compared with a median of 4.56 (2.78–6.13) minutes recorded in longitudinal operational logs prior to full system rollout; median transport completion time was 24.3 (13.41–32.87) minutes compared with 45.5 (30.74–58.32) minutes previously (Table 1). As detailed in Sections 2.1 and 2.5, the pre-implementation figures are drawn from incomplete, non-systematically sampled manual records and are reported descriptively rather than as a formally matched statistical comparator; consequently, no inferential before–after statistics are presented and the values should be interpreted descriptively only. Within the post-implementation period, routine specimen-transport turnaround was approximately 50% shorter than described in prior workflow records, although this comparison is descriptive and not based on a standardized pre-implementation dataset, and daily manual (telephone-based) dispatch requests decreased by 34.2%, corresponding to a qualitative reduction in coordination burden between clinical departments and the transportation team.

**Table 1:** Dispatch time and transport completion time, pre- vs. post-implementation reference values.

Period	Dispatch time, min, median (IQR)	Completion time, min, median (IQR)
Pre-implementation (historical reference; non-systematic manual records)*	4.56 (2.78–6.13)	45.5 (30.74–58.32)
Post-implementation (Jan 1–Jun 30, 2026; system-recorded)	0.57 (0.31–0.82)	24.3 (13.41–32.87)

*Pre-implementation values are historical reference figures from incomplete, non-systematically sampled manual records. They are provided for descriptive/contextual comparison only and are not a matched control dataset; therefore, no formal before–after statistical inference is reported.

### 3.2 Time-of-day task-volume distribution

System records revealed a distinct bimodal daily workload pattern, with two clear peak periods (07:00–11:00 and 13:00–17:00, each reaching task volumes in the tens of thousands) and low-volume periods overnight (00,00–06:00) and in the evening (20,00–24:00). Based on this time-stratified pattern from the January–June 2026 data, management can identify peak and trough windows and their approximate magnitude to inform dynamic staff scheduling—reinforcing staffing during peak periods and consolidating on-duty staff during low-demand periods—potentially reducing the efficiency losses and cost associated with uneven staff-task matching under a fixed-shift model (Figure 3).

**Figure 3:**
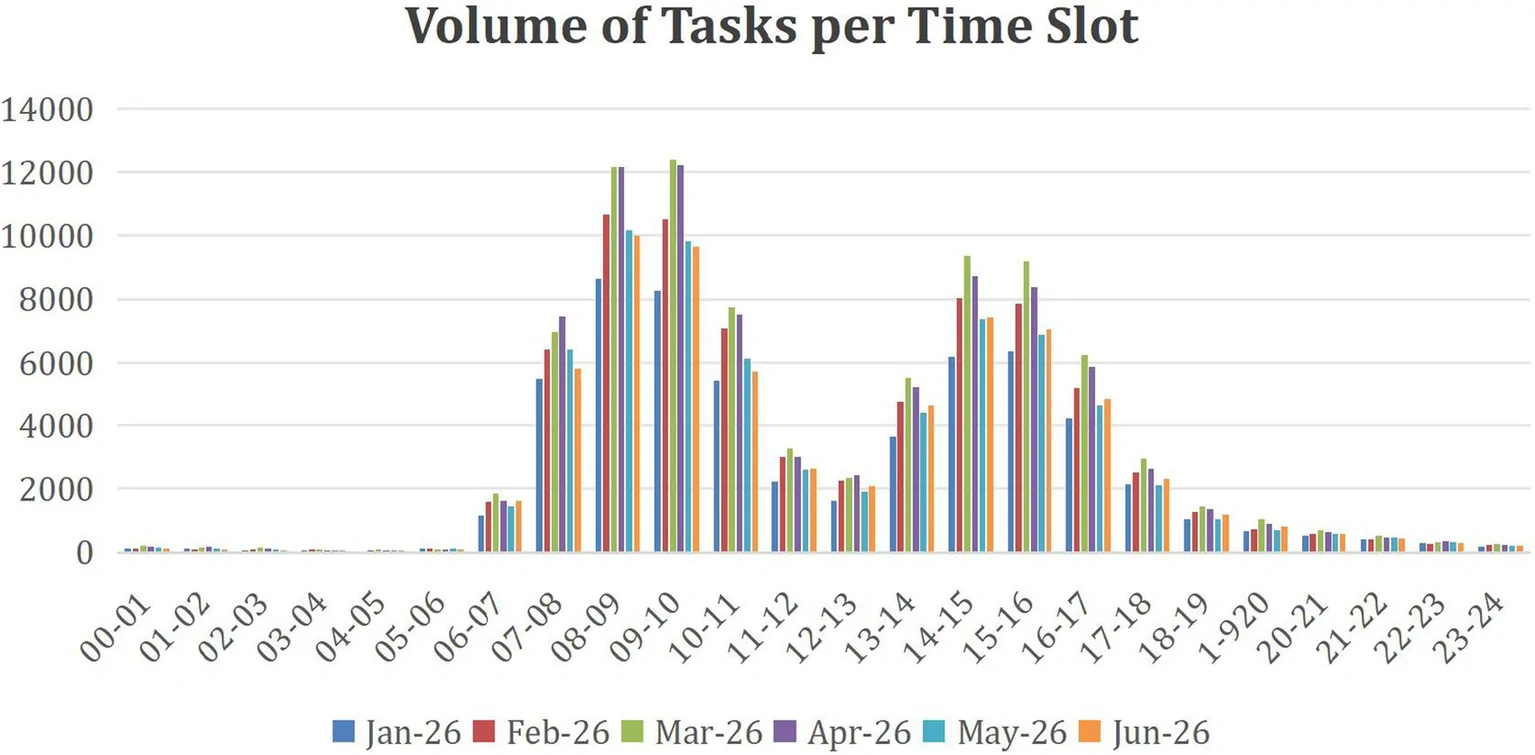
Analysis of transportation task time periods.

### 3.3 Transport-tool utilization

Across three core scenarios (surgical escort, imaging examination transport, and ward-transfer/discharge transport) between January and June 2026, 24,545 valid transport-tool usage records were analyzed (data completeness 100%). Transport-tool selection showed clear stratification: independent ambulation (54.5%) and wheelchair transport (32.0%) together accounted for 86.5% of transports, while stretcher (6.5%) and bed transfer (7.0%) accounted for the remaining 13.5% and were concentrated in high-acuity, bed-bound patients requiring cross-departmental transfer (Table 2). Exploratory clinical stratification suggested that independent ambulation was used predominantly for routine examinations and admission/discharge in low-acuity patients; wheelchair transport was concentrated among older adult(s) with chronic conditions or limited mobility, frequently for same-day surgical escort; and stretcher/bed transfer was concentrated in operating-room and intensive-care-unit transfers of critically ill, bed-bound patients. This distribution provides a descriptive profile of institutional transport demand that may inform dynamic tool pre-positioning during peak periods, although we did not formally test for statistical differences across scenarios in tool-selection proportions in this report.

**Table 2:** Transport-tool utilization, January–June 2026 (*N* = 24,545).

Transport tool	*n*	%
Independent ambulation	13,377	54.5
Wheelchair	7,854	32.0
Bed transfer	1,718	7.0
Stretcher	1,596	6.5
Total	**24,545**	**100.0**

*Percentages calculated from *n* reported in the original dataset (54.5%, 32.0%, 7.0%, and 6.5% of *N* = 24,545); values are rounded to one decimal place and may not sum to exactly 100.0% due to rounding. Bold number indicate the total sample size of transport tasks.

### 3.4 Patient satisfaction

Using the hospital’s satisfaction-survey platform, 5,508 inpatients completed a structured questionnaire assessing staff appearance, staff attitude, identity-verification practice, staff attentiveness, and responsiveness/assistance (0–100-point scale per item), administered between January and June 2026. Overall satisfaction with the central transportation service was 97.78% (proportion of respondents scoring ≥80 on the overall item); item-level satisfaction rates ranged from 95.51% (staff attentiveness, the lowest-scoring item) to 98.14% (staff appearance, the highest-scoring item) (Table 3). No comparable pre-implementation satisfaction survey dataset was available for formal statistical comparison; this cross-sectional post-implementation result is therefore reported descriptively.

**Table 3:** Inpatient satisfaction with central transportation services (*N* = 5,508).

Survey item	Satisfaction rate (%)
Staff appearance	98.14
Staff attitude	97.91
Identity verification practice	97.95
Staff attentiveness	95.51
Responsiveness to questions	97.62
Assistance provided	97.70
Overall satisfaction	**97.78**

Bold value represents the overall inpatient satisfaction rate.

## 4 Discussion

### 4.1 Algorithm-driven scheduling and transport efficiency

Within the post-implementation dataset, algorithm-driven dispatch was associated with markedly shorter task-response and transport-completion times compared with the historical, manually dispatched workflow. Under the traditional model, dispatchers relied on manual matching and telephone confirmation, a process associated with information loss and communication inefficiency. Automated data capture from HIS/LIS, combined with real-time location and workload data, enabled near-immediate dispatch and multi-task consolidation, which is a plausible mechanistic explanation—though not, on the basis of this retrospective descriptive design, a formally demonstrated causal pathway—for the observed reduction in emergency-transport time. This pattern is consistent with prior real-world evaluations of IoT-based hospital dispatch (10, 12): a real-time-tracking, data-driven dispatch system implemented at a Taiwanese hospital similarly reduced overtime delays in patient transport relative to traditional manual dispatch, and broader work on IoT-enabled logistics dispatching has argued that integrating sensing, identification, and decision-support technology can improve the analytic, decision-making, and execution capability of a logistics system (12, 16).

### 4.2 End-to-end traceability and patient-safety implications

Traceability is a foundational requirement of logistics-related patient safety. Under the traditional model, the absence of real-time tracking made root-cause investigation of specimen loss or mis-delivery difficult. The barcode-verification and electronic-ledger workflow implemented here requires dual identity/item confirmation at each handoff step, which is intended to support—rather than guarantee—more consistent operator behavior. More broadly, RFID/IoT-based identity and location verification has been proposed elsewhere (7, 8, 17) as a mechanism to reduce specimen- and patient-identification error in hospital logistics; we frame our own barcode-verification observations as consistent with, rather than confirmatory of, this broader literature.

### 4.3 Data-driven resource and staffing management

The stratified pattern of transport-tool utilization (86.5% low-acuity/ambulatory-capable vs. 13.5% high-acuity/bed-bound) provides a descriptive basis for equipment provisioning and staff scheduling (18). Under a traditional “uniform provisioning” model, equipment shortages during peak periods and idle assets during trough periods are common. A time-of-day/tool-utilization mapping of the kind presented here could, in principle, support dynamic pre-positioning of wheelchairs and stretchers during predictable peak windows, though we did not prospectively test whether implementing such a policy changed asset utilization or wait times, and we present this as a hypothesis for future evaluation rather than a demonstrated outcome.

Reported workloads are broadly consistent in scale with those described in comparable tertiary-hospital transportation information-system evaluations (13, 14). Jiang et al. (13) describe an intelligent inpatient transportation system implemented in a large tertiary hospital, while Chen et al. (14) report operational experience with a hospital central-transport dispatch information system. Because the populations, observation windows, definitions, and data-collection methods differ across studies, these publications are used only to contextualize the scale and implementation setting, not as quantitative validation of the present workload estimates.

### 4.4 Patient and staff experience

The satisfaction results reported here are broadly consistent with the premise that transparent, trackable transport processes can reduce patient-perceived waiting-related uncertainty and reduce the coordination burden on clinical staff who would otherwise need to repeatedly follow up on delayed transports. This is consistent with a growing literature describing digital transformation—including IoT, AI, and big-data-enabled process optimization aimed at improving real-time decision-making and patient safety (16, 17, 19, 20)—as a component of broader efforts to improve perceived service quality in hospitals; we note, however, that our satisfaction data are cross-sectional and post-implementation only, and cannot by themselves establish that satisfaction improved as a result of the system.

### 4.5 Considerations on workforce efficiency under institutional expansion

In 2025, the hospital completed a multi-campus expansion, increasing bed capacity from 4,300 to 4,900 (+13.95%) across three campuses spanning more than 20 km, which increased demand for cross-campus specimen and patient transport. Under a traditional linear staffing model in which additional beds are matched with proportionally added transport staff, this scale of expansion would ordinarily require a substantial increase in headcount. In this setting, the existing 334-member transportation workforce absorbed the expanded task volume without a corresponding formal increase in staffing establishment during the observation period. We present this as a descriptive operational observation rather than a formal cost-effectiveness analysis: we did not conduct a prospective, itemized accounting of marginal staffing costs avoided, nor did we control for concurrent operational changes (e.g., process redesign, shift restructuring) that may have contributed independently to this outcome. A more rigorous prospective or comparative economic evaluation is warranted before quantitative cost-savings estimates are reported.

This observational pattern is in line with reports from comparable hospital logistics information systems, where digital dispatch platforms have been associated with maintained or improved service capacity despite increasing institutional scale (14, 19).

### 4.6 Limitations

Several limitations should be considered when interpreting these findings. First, this is a single-center, retrospective, descriptive evaluation without a rigorously matched pre-implementation control period; the pre/post figures in Table 1 derive from incomplete, non-systematically sampled historical records and should not be interpreted as confirmatory evidence of a causal system effect. Second, we did not adjust for concurrent confounders such as seasonal volume variation, the 2025 multi-campus expansion, or concurrent process changes. Third, the analysis focused on operational efficiency and satisfaction and did not assess downstream clinical outcomes such as length of stay or laboratory turnaround time. Fourth, adverse events relied on voluntary staff reporting and may therefore be under-ascertained; staff reluctance to report errors or near-misses could lower the observed event count, while changes in reporting culture after implementation could alter ascertainment independently of the underlying event rate. Consequently, the observed adverse-event counts should be treated as a lower-bound signal rather than a complete incidence estimate, and any temporal comparison would be vulnerable to ascertainment and reporting-culture bias (21). Fifth, real-time patient-facing progress tracking is not yet implemented. Finally, the adaptive scheduling algorithm has not been evaluated under sudden, large-scale task surges (Figures 4, 5).

**Figure 4:**
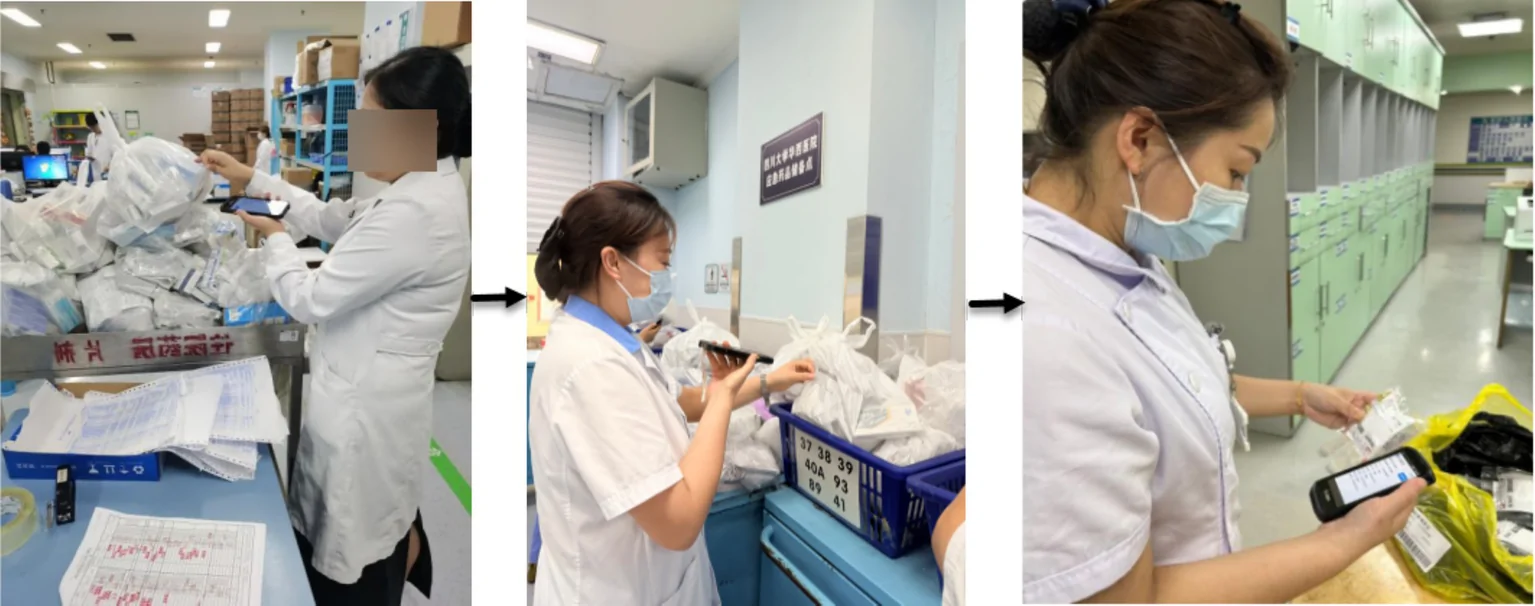
Pharmacy staff-transport staff-ward nurses scan medication labels using handheld PDAsatthehandover point.

**Figure 5:**
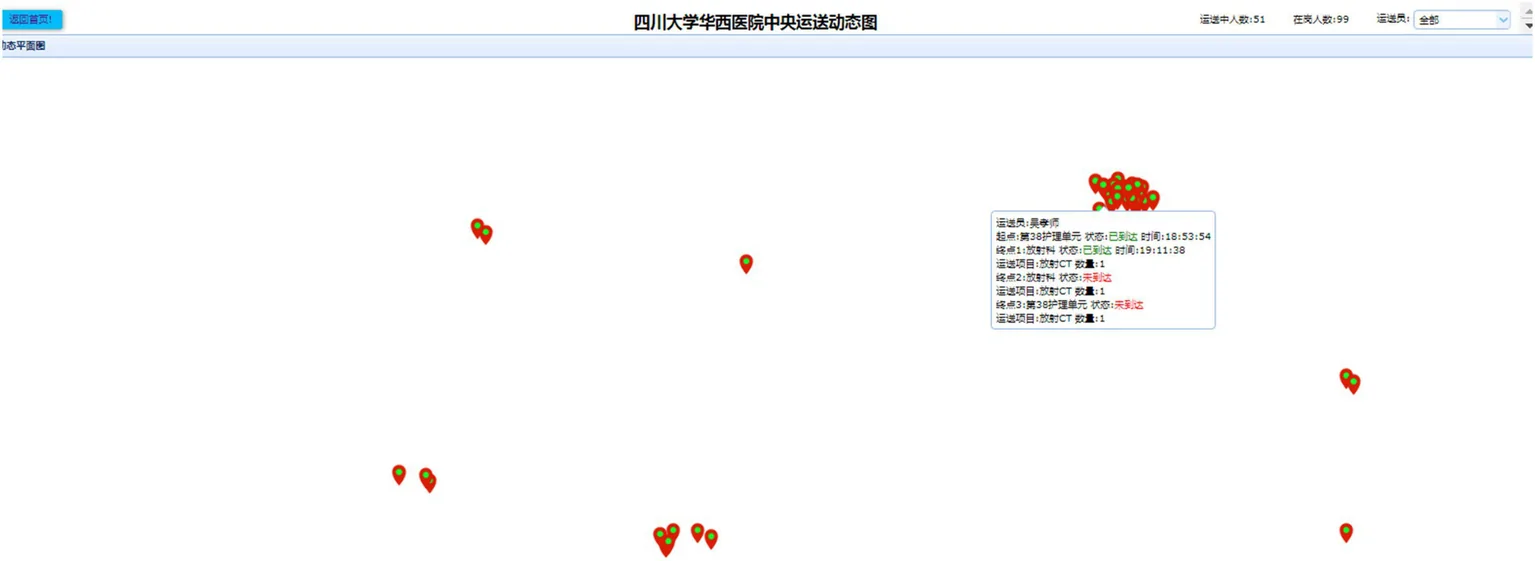
Photograph or de-identified screenshot of the central transportation dispatch dashboard showing real-time task status and staff locations.

The machine-learning-assisted prediction and optimization literature provides a broader methodological perspective for future scheduling refinement. Recent work has examined machine-learning-assisted multi-objective optimization for engineering decision support, including Gaussian-process/support-vector prediction combined with multi-objective genetic optimization in robotic mechanical design (22). In healthcare IoT, federated-learning approaches have also been reviewed as a means of distributed anomaly detection under privacy constraints (23). These studies are methodological analogues rather than direct evidence for the present dispatch system; the current study did not deploy machine learning or federated learning, and future work would need prospective validation before such methods are incorporated into clinical-logistics scheduling.

## 5 Conclusion

We describe the design and post-implementation operational performance of an intelligent centralized transportation dispatching information system, integrating RFID, IoT, and an intelligent scheduling algorithm, deployed across four core logistics scenarios in a large tertiary hospital. During the observed six-month period, the system was associated with short dispatch and transport times, a stratified and interpretable pattern of transport-tool utilization, and high patient-reported satisfaction. Given the retrospective, single-arm, descriptive nature of this evaluation and the absence of a rigorously matched baseline period, these findings should be regarded as a real-world operational description rather than causal evidence of system effectiveness. We recommend that future evaluations adopt a prospectively defined baseline or interrupted-time-series design, incorporate a formally denominated safety/complaint-rate metric, and examine associations with downstream clinical indicators such as LOS and TAT, to more rigorously establish the incremental value of intelligent transportation dispatching for large-hospital logistics management.

## Data Availability

The raw data supporting the conclusions of this article will be made available by the authors, without undue reservation.
